# Tapping the Potential of Resilience to Support an Integrated and Person-Centred Approach to Health and Wellbeing—Developing a Simple Assessment Tool for Practice

**DOI:** 10.3390/ijerph19052679

**Published:** 2022-02-25

**Authors:** Katharina Viktoria Stein, Wolfgang Rutz, Birgit Hladschik-Kermer, Thomas E. Dorner

**Affiliations:** 1 Karl Landsteiner Institute for Health Services Research, 3454 Sitzenberg-Reidling, Austria; thomas.dorner@bvaeb.at; 2 Social Insurance Fund for Public Service, Railway and Mining Industries, 1080 Vienna, Austria; 3 Department for Rehabilitation Research, Karolinska Institutet, 17177 Stockholm, Sweden; rutz.wolfgang@gmail.com; 4 Centre for Multidisciplinary Research on Religion and Society, Uppsala University, 75120 Uppsala, Sweden; 5 Department of Social Work and Health, University of Applied Sciences Coburg, 96450 Coburg, Germany; 6 Department of Medical Psychology, Medical University, 1090 Vienna, Austria; birgit.hladschik-kermer@meduniwien.ac.at

**Keywords:** resilience, person-centred care, integrated care, assessment tool, social determinants of health, mental health

## Abstract

(1) Background: The push towards population health management and the need for new approaches in health services delivery focusing on the prevention and management of chronic diseases has helped in advocating for more person-centred care, and thus for integration of physical and mental health. Resilience plays a key role in supporting sustainable lifestyle changes and promoting health and wellbeing, but most assessment tools available today are too long for widespread use. The purpose of this paper is to describe the development of a new diagnostic tool to capture a person’s resilience and resources. (2) Methods: This paper outlines the interrelatedness of different theories of salutogenesis, social determinants of health and health promotion with resilience and establishes resilience as a key enabler to promote health and wellbeing. (3) Results: A new, short questionnaire is proposed based on the triade of evidence-based medicine, which should be easy to use and give a good assessment of a person’s resilience. (4) Conclusions: There are many reasons why the call for a short and easy-to-use assessment tool for resilience is warranted. In view of the international transition towards integrated, person-centred health systems, such a tool would find many usages. It would also support the strategies to tackle multi-morbidity, complex conditions and the social determinants of health in its focus on strengthening an individual’s ability to cope with adverse events, and actively engage in health promotion and community involvement programmes. The next step is to test the tool in practice and validate it.

## 1. Introduction

In the wake of a more holistic understanding of health and wellbeing and the growing recognition of the complex determinants of health [1,2,3], the attention has shifted towards understanding the interplay between physical and mental health better [4,5,6]. While the biopsychosocial model of health [7] is anything but new, modern health systems have been very slow in adapting their assessment and service delivery processes to these complexities. The push towards population health management and the need for new approaches focusing on the prevention and management of chronic diseases has helped in advocating for more person-centred care and thus for integration of physical and mental health [5,8,9,10]. The interaction between physical and mental health, either reinforcing good outcomes or exacerbating bad ones, has been demonstrated for a plethora of diseases and risk factors [11,12,13,14].

Resilience is a key concept supporting these efforts, as it offers a counterweight to the deficit-oriented, disease-focused and reactive approach to health service delivery, which prevails in most systems to this day. While there is a plethora of literature available on the concept, and various theories and models make use of it, it is underutilised in clinical and health promotive settings. This is partly due to the fact that most assessment tools and applications are both very long and do not cover all dimensions of the concept.

Based on these reflections, the purpose of this paper is to suggest a new brief diagnostic tool to capture a person’s resilience and resources. It is based on existing resilience questionnaires but places brevity and ease of application at the centre. As such, it is intended to be used in all settings and by any professional, care team, or layperson who wants to adopt a person-centred approach and assess or self-assess the resilience of a person.

## 2. Theoretical Underpinnings—Setting the Stage for Resilience (Material and Methods)

### 2.1. The Concept of Salutogenesis

**Figure 1:**
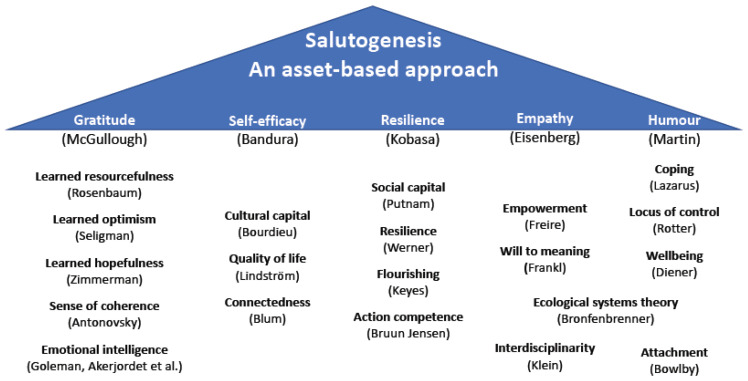
The concepts and theories of salutogenesis. Source: Own illustration, based on [6,16].

Aron Antonovsky (1979) first described the concept of salutogenesis as opposed to pathogenesis as the need to emphasise the relationship between body and mind of an individual in the context of their social environment in order to successfully treat and support a person [15]. He described the importance of looking at the resources and assets of a person, both internally and externally, to understand their ability to cope with adverse events and consequently to lead a fulfilled and healthy life. His works gave rise to a plethora of asset-based approaches, some of which are illustrated in Figure 1. In their seminal work to summarise the broad scope of theories that explore salutogenesis, Lindström and Eriksson (2010) [6,16] describe concepts from as varied a range of fields as psychology, philosophy or economics, but all look at the strengthening and empowerment of the person in interaction with their environment.

In their articles, Lindström and Eriksson organised the various concepts, theories and models under five headings, four of which are briefly described below. As the main focus of this paper is on resilience, it is discussed in more detail in Section 2.3.

#### 2.1.1. Gratitude

McGullough et al. [17] conducted four studies in the USA to explore gratitude as an affective trait and how it influences a person’s relationships, attitudes towards others, and spirituality. Importantly for the discussion around health and wellbeing, they found that more grateful people tend to experience better health and wellbeing, are less prone to depression and anxiety, and demonstrate empathy and supportive behaviour more often than their less grateful contemporaries. The results are supported by other concepts, such as Antonovsky’s Sense of Coherence [18].

#### 2.1.2. Self-Efficacy

Bandura defines self-efficacy as a person’s belief in their capabilities to exercise control over their own functioning and over events that affect their lives [19]. The concept underlines the importance of an individual’s perceived role in a community, the importance of role models for one’s own development and the negative feedback loop of ill health, low self-efficacy and the ability to cope. At the other end of the line, concepts such as Seligman’s Learned helplessness [20] describe the inability of people to act or react to an event, whether adverse or merely overwhelming, if they feel it is out of their control.

#### 2.1.3. Empathy

Eisenberg and colleagues first started to explore the relationship between empathy and social behavior in the 1980s and 90s, e.g., [21] linking a more empathetic person with higher social competence, altruism and social engagement, among other things. This, in turn, leads to stronger and more positive emotions, which amplify resilience and coping mechanisms when adversity is faced. Positive emotions further contribute to better physical and mental health, improving the general health and wellbeing of empathetic people.

#### 2.1.4. Humour

The German catchphrase “Laughing makes healthy” is evidenced by science, mainly through the research conducted by Martin and colleagues over the past three decades [22]. Similar to the concept of empathy, humour can contribute to better overall health and wellbeing through a more light-hearted approach to life in general, but also to negative experiences, especially stress. Different styles of humour can influence the way we interact, keep up relationships and influence our overall health and wellbeing.

All of these concepts stress the interrelationship of body and mind, as well as the question around an individuals’ perceived and actual position in and interaction with their community and society. While there may be differing opinions as to the categorisation of the concepts under Lindström and Eriksson’s umbrella of salutogenesis, it provides a very useful overview of the many inter-related concepts influencing, supporting and aligning with health and wellbeing.

### 2.2. The Social Determinants of Health and the Influence of Mental Health

As with the concept of salutogenesis, the social determinants of health have become a cornerstone of understanding how health and wellbeing are influenced by many factors, both internal and external. It was first described by Marmot and colleagues during the groundbreaking Whitehall I [23] and II studies [24], which focused on English civil servants in the Whitehall district of London. The district is home to the UK parliament and government, and it was expected that the primarily white, male, well-educated and affluent civil servants working there would all have very similar health outcomes. Surprisingly for them, the researchers found a “social gradient”, which clearly showed that health outcomes depended heavily on the social status, hierarchical level and personal influence and power the civil servants had. Building on these findings, Marmot and colleagues developed the concept further, and the WHO illustrated how other sectors such as education, social environment, infrastructure and transport heavily influence the health status and health outcomes of individuals and communities [2]. Social determinants of health are broadly defined by WHO as “non-medical factors that influence health outcomes. They are the conditions in which people are born, grow, work, live and age, and the wider set of forces and systems shaping the conditions of daily life. These forces and systems include economic policies and systems, development agendas, social norms, social policies and political systems.” [25]. There is no finite list as the concept and understanding expand, but it is clear that taken together, they play a much bigger part in influencing health and wellbeing than the health system and access to care itself [2,3].

Social determinants are linked closely with different concepts of coping, self-efficacy or learned helplessness, as they influence an individuals’ capacity of taking control of one’s own life, of interacting with others in the community and finding one’s place, and vice versa. Even before the WHO report on the social determinants of health (2008) [2], this was highlighted in the WHR 2001 on the importance of mental health [4]. Together these key documents underline the importance of taking a person-centred and holistic approach towards health and wellbeing and prepare the ground for a wider application of resilience in everyday health and care.

### 2.3. Taking a Closer Look at Resilience—A Definition

Resilience deals with the question of how people react to stressful events in the moment and over time. It is defined as “the process of adapting well in the face of adversity, trauma, tragedy, threats, or even significant sources of stress—such as family and relationship problems, serious health problems, or workplace and financial stressors.” [26]. It can thus be circumscribed as a person’s ability to “bounce back” from difficult experiences, and maintain a sense of who they are [27]. Resilience enables a person to not only deal with powerful emotions but to actively manage them and draw conclusions as to how to deal with them. It comes from a sense of trust in one’s social network and oneself and empowers a person to communicate one’s emotions to seek support and help. As Kobasa and Maddi described it [28], resilience thus is a combination of cognitive appraisal, behavioural coping, social resources and health-promoting behaviours. Kobasa’s three Cs of Hardiness [29] closely align with her description of resilience and reinforce the concept, as is illustrated in Table 1.

Since then, resilience has been the focus of much research, and various tools have been developed to assess it see for example [32].

### 2.4. A Review of Resilience Assessment Tools

There are a plethora of resilience assessment tools available, many of which are neither validated nor published in the scientific literature. A review of nineteen resilience measures [32] found only three with above-average psychometric ratings: CD-RISC, BRS and RSA. These are described in more detail below, in addition to the Resilience Scales, which are still used widely and have been validated in several languages.

#### 2.4.1. Resilience Scale

The Resilience Scale was developed and validated by Wagnild and Young in 1993 [33] with a sample of older adults (aged 53 to 95 years). The 25 items of the scale positively correlate with physical health, morale and life satisfaction, but not with depression. The scale uses five characteristics to measure resilience: Meaningful Life (or Purpose), Perseverance, Self-Reliance, Equanimity and Existential Aloneness. Two subscales, the 17-item Personal Competence subscale and the 8-item Acceptance of Self and Life subscale, are used to examine the five characteristics. A renewed validation of the scale in 2009 [34] confirmed its internal consistency and construct validity, which reaffirmed its continued usefulness as a tool to assess resilience.

#### 2.4.2. Connor–Davidson Resilience Scale (CD-RISC)

Connor–Davidson originally developed this scale as a self-report measure of resilience for people with Post Traumatic Stress Disorder (PTSD) [35]. There are validated versions of 2, 10 and 25 items that measure resilience as a function of five interrelated components: Personal Competence, Acceptance of Change and Secure Relationships, Trust/Tolerance/Strengthening Effects of Stress, Control and Spiritual Influences. The instrument is widely recognised, and many studies use it with a wide range of populations. As such, CD-RISC is considered a higher scoring scale in the psychometric evaluation of resilience [32].

#### 2.4.3. Resilience Scale for Adults (RSA)

The RSA is another self-report scale for adults [36]. It is recommended for use in the fields of health promotion and clinical psychology. It is another scale that uses five scoring items that address the intrapersonal and interpersonal protective factors. The items are called Personal Competence, Social Competence, Social Support, Family Coherence and Personal Structure. The authors identified highly resilient individuals as those with family support and cohesion, external support systems and dispositional attitudes and behaviours. A later study [37] used the RSA to measure the relationship between personality, intelligence and resilience. The authors established many links between personality and resilience factors, but the study did not yield any significant results related to cognitive ability [37]. These findings were confirmed by Windle et al. [32], who concluded that the RSA’s strength lies in assessing protective factors against psychological disorders.

#### 2.4.4. Brief Resilience Scale (BRS)

The Brief Resilience Scale (BRS) [38] is a self-rating questionnaire aimed at measuring an individuals’ ability to cope with stress. Even though it has so far not been used in a clinical population, it has the potential to provide insights into individuals with health-related stress. The BRS instrument consists of three positively worded items and three negatively worded ones [39]. All six measure a persons’ ability to cope with adversity. During the development process, protective factors such as social support were controlled for in order to obtain a reliable resilience measure [38].

In summary, the four validated instruments discussed in Table 2 were successfully applied across a variety of populations, but only the Resilience Scale (RS) and the Resilience Scale for Adults (RSA) specifically focus on prevention, and only the RS and the CD-RISC were tested for the general public. All three of them are also quite long, while the Brief Resilience Scale (BRS) has a narrow focus on coping with stress in very specific sub-populations. It was, therefore, felt appropriate to develop a new assessment tool, which combines the applicability in the general public (CD-RISC, RS) for health promotion and prevention efforts (RS, RSA) with the briefness of the BRS.

## 3. The Need for a New Assessment Tool (Results)

Resilience in its broadest form, as outlined above, has a great potential to help mitigate the challenges and developments of the 21st century. As it encompasses personal, community and societal aspects, it is strongly influenced by social determinants of health, and thus, the movement towards integrated, people-centred systems [40], which focuses on building healthy communities and neighbourhoods and would greatly profit from incorporating the concept into their toolkit. From the often discussed ageing populations and the rise of complex needs to the often ignored issues around flight and migration, transculturation or the impact of the financial crisis of 2008 on societies and public services, to climate change and the advances of new technologies and social media, to ultimately the still largely unknown long-term consequences of the COVID-19 pandemic—all of these complex issues influence the health and wellbeing of individuals and populations, on top of causing high levels of stress. The interrelationships and complexities are governing these issues individually and on an aggregate level call for a completely new approach to assessing their impact on the health and wellbeing of individuals and communities. These need to be innovative, inter-disciplinary and multi-factorial, and take into account different population needs as well as being based on a person-centred approach [4,10,41].

Thus, it has become ever more apparent that traditional clinically driven assessments fall short in capturing the complex interactions and interrelationships between physical health, mental health, socio-economic and behavioural factors. This has further been illuminated by the current COVID-19 pandemic, which disproportionately affected vulnerable communities, such as immigrants, ethnic minorities, older people or people living in poor neighbourhoods [41,42,43,44]. These populations not only have poorer physical health outcomes, but they also have fewer coping mechanisms and resources at their disposal to adapt their behaviours and strengthen their mental health [45,46]. When faced with a crisis, such as the current one, these inequalities impede access to adequate care and exacerbate already poor outcomes [41,42,44,46]. The calls for more people-centred integrated health systems have therefore grown louder during the pandemic, and models, which already follow the principles of person-centred care, have fared better in addressing the needs during the crisis [41,47]. In order to take full advantage of the potential of resilience within person-centred health promotion and strengthening approach, a short, simple and reliable tool is needed, which can be used by anyone in any setting.

### 3.1. Proposing Four Domains to Assess Resilience

By synthesising the concepts and dimensions described above, several domains emerge, which are generally included in the assessment of a person’s resilience and their capacity to adjust or react to adversity, whether physical, mental or social. As was shown, all of the concepts are closely related and sometimes used to describe one another, which makes a clear distinction difficult. This is further exacerbated by the length of the existing assessment tools and the lack of reliability in many. In the best tradition of evidence-based medicine, which combines long-standing experience in practice with scientific evidence and patient values and preferences [48], the following four domains were developed based on a review of the literature and experience in practice as the basis for a simple assessment tool. We stipulate that the four domains “Meaning”, “Social connectedness”, “Self-determination” and “Dignified identity” cover different dimensions of resilience while at the same time ensuring independence from one another.

**Figure 2:**
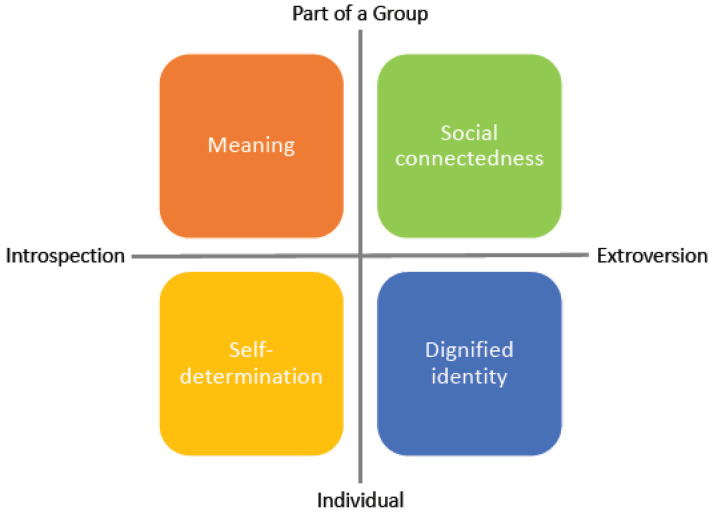
Four domains to assess resilience.

The four domains can be summarised on either “looking in”, i.e., the perception of oneself (Self-determination) and of one’s position in relation to others (Meaning); or as “looking out”, i.e., the experience of the individual (Dignified identity) as being part of a group (Social connectedness). In other words, Figure 2 depicts the four domains and the perspective they represent: on the one hand, the individual reflecting on themselves (individual and introspective = Self-determination) and their experience in the world (individual and extroverted = Dignified identity); on the other hand the domains represent the relationship between the individual and the group (Meaning and Social connectedness). The four domains can further be described thus:

*Self-determination =* Being in charge of one’s own life, having possibilities of influencing one’s own living conditions, not being helpless. This domain is closely related to Kobasa’s Control and Behavioural (transformational) Coping and captures the concepts of Bandura’s self-efficacy and Seligman’s positive psychology;

*Dignified identity =* To be treated as an individual in one’s own right, with personal identity and human rights, not to feel humiliated, to be respected as a valued member of society. This domain represents the inalienable integrity of a person’s physical, mental and social health and wellbeing. It thus relates to Antonovsky’s Sense of Coherence;

*Meaning =* The feeling of an “over-individual” cohesion, including in an existential and spiritual context, to feel part of a community, a workplace. This domain connects to the concept of gratitude and also aligns with Frankl’s search for meaning in life through the realisation of values;

*Social connectedness =* The experience of love, to be cared for, but also to be able to help others, to feel socially relevant. This domain is closely related to empathy and to Kobasa’s Commitment and Social resources and health-promoting behaviours.

### 3.2. Developing a Simple Assessment Tool to Measure Resilience: The Human Condition-4—Satisfaction Index

It is stipulated that these four domains cover a sufficient number of dimensions and the broad scope of the concept of resilience to be the basis for the development of a brief and easy-to-use assessment tool. The four domains outlined above are expected to represent an accurate picture of a person’s ability to stay healthy, change one’s life and lifestyle and reduce the risk of mental illness or disorder. They also enable a more holistic, person-centred approach to co-design adequate support services and possible health plans. In order to turn them into an easy-to-use assessment tool, the principle of the WHO-5 wellbeing scale [49] was used as a guide. For each domain, one question was formulated, and a six-part Likert scale was developed to evaluate the individual scores for each of the four domains. Statement 1 represents Self-determination, Statement 2 relates to Social connectedness, Statement 3 reflects Meaning and Statement 4 is Dignified identity. Table 3 represents the assessment tool.

The scores are added up to receive an overall estimate of one’s resilience: 0–5 low, 6–15 intermediate, 16–20 high. Based on the scores, the evaluator and the person should discuss each of the four domains in more detail and analyse the individual’s internal and external resources, barriers and assets.

The overall goal of the self-assessment and subsequent conversation is to develop a co-designed, person-centred health plan, which enables the person to strengthen their resources and, ultimately, resilience. Example questions can be used to guide the conversation and discussion:

- What is the individual picture and phenomenology of your challenges/barriers, and what consequences did they have or will they have in your life?
- What abilities to change can you identify, and which of them are realistic and feasible for you to change with our assistance?
- How can your socio-psychological and living environment be characterised?
- How is your physical and mental situation characterised today?
- By your hereditary and family history, by your social and familiar environment, your working place, your existential situation, your beliefs, values and ideologies?
- How can changes be made and improvements realised?

Based on this comprehensive analysis, a shared health plan can be developed to support the individual in their endeavour to strengthen resilience and improve their overall health and wellbeing.

## 4. Next Steps of Development

The assessment tool forms part of a comprehensive evaluation and diagnostic suite of instruments, which are used in an innovative, new health promotion facility of the Austrian Social Insurance Fund for Public Service, Railway and Mining Industries [8]. This facility is aimed at people of working age who want to improve their overall health and wellbeing, irrespective of existing risk factors or diseases. The three-week programme, which is split into a two-week foundational part and a follow-up week three months later, offers activities in five areas: physical activity, nutrition, mental health, health literacy and social capital. The suite of assessment tools and clinical diagnostics supports the tailoring of the activities to create individualised programmes according to the needs and preferences of the participants.

In order to determine the usability and validity of the tool, it will be tested in practice. A first step will be to determine whether the four dimensions illustrated in Figure 2 are in actual fact independent. The Human Condition-4—Satisfaction Index will be used alongside the WHO-5 [49], WHO-Quol-Bref [50], the Perceived Stress Questionnaire [51] and the validated German version of the Resilience scale [52]. By using a sample of the first 500 participants, a factor analysis with every item of the different questionnaires will determine which dimensions are actually measured. Reliability will be measured using Cronbach’s alpha to determine inter-relatedness between the items in each dimension. If the above-determined domains are represented by the dimensions, this will be followed up with a validation study.

The overarching goal is to facilitate the utilisation of resilience as part of an integrated, person-centred approach in any setting by building on a person’s individual capacity and co-designing a plan of support and care that is driven by the person. The key strength of the proposed assessment tool lies in its simplicity and brevity.

## 5. Conclusions

There are many reasons why the call for a short and easy-to-use assessment tool for resilience is warranted. In view of the international transition towards integrated, person-centred health systems, which put healthy individuals and communities at the centre of care, such a tool would find many usages. It would also support the strategies to tackle multi-morbidity, complex conditions and the social determinants of health in its focus on strengthening an individuals’ ability to cope with adverse events and actively engage in health promotion and community involvement programmes. As with all these tools, limitations primarily concern the definition of the domains and whether they can actually be assessed by the questions formulated. Another concern may be raised by the unconventional development of the tool. Nevertheless, this was led by the triade of evidence-based medicine [48], combining a long-standing experience in both psychiatric practices, as well as in health promotion and public health programmes, with a thorough grounding in theoretical concepts and guided by the principles of person-centred care. We think it is high time for resilience to come into its own and play a key role in addressing the manifold challenges individuals face today. As this is a work in progress, comments and feedback are actively sought and very welcome.

## Figures and Tables

**Table 1:** Comparison of Kobasa’s Three Cs of Hardiness [29] with her Resiliency Mechanisms [28,30,31].

Three Cs of Hardiness	Resiliency Mechanisms
*Control =* the tendency to believe and act as if one can influence the events taking place around oneself through one’s own efforts	*Behavioural (transformational) coping* = optimistic style of transforming stressful events into less stressful ones
*Commitment =* the tendency to involve oneself in the activities in life and having a genuine interest in and curiosity about the surrounding world	*Social resources and health-promoting behaviours =* recruiting and making adequate use of social resources
*Challenge =* the belief that change rather than stability is the norm in life (growth versus fixed mindset)	*Cognitive (optimistic) appraisal =* putting stressful circumstances into perspective and interpreting them in a less threatening manner

Source: Own table, based on [28,29,30,31].

**Table 2:** Overview of resilience assessment tools.

	Connor–Davidson Resilience Scale (CD-RISC)	Resilience Scale (RS)	Resilience Scale for Adults (RSA)	Brief Resilience Scale (BRS)
Items	25	25 or 14	37	6
Focus	Diagnosis and therapy of PTSD	Prevention Identification of people at risk	Prevention Monitoring	Shortness Stress resistance
Definition of resilience	Resilience as a changeable entity, which indicates health status	Resilience as protective personality trait		Resilience as capability to quickly recuperate from stressors and come back on one’s feet
Dimensions	Personal Competence Acceptance of Change and Secure Relationships Trust/Tolerance/Strengthening Effects of Stress Control Spiritual Influences	Acceptance of self Personal competence	Personal Competence Social Competence Social Support Family Coherence Personal Structure	Ability to bounce back from adversity
Tested in the following sample populations	General public Patients in primary care Patients with generalised anxiety disorder	Older adults	Patients with mental disorders General public	Students Patients in cardio-vascular rehab People with chronic pain
Scale	5-part (0–4)	7-part	4-part	6-part (1–5)

**Table 3:** The Human Condition-4—Satisfaction Index.

	All of the Time	Most of the Time	More than Half of the Time	Less than Half of the Time	Some of the Time	At No Time at All
1. I have felt self-directed and autonomous—“in charge” of my life.	5	4	3	2	1	0
2. I have felt that people care for me and that there are persons I care for.	5	4	3	2	1	0
3. I have felt that my life is meaningful.	5	4	3	2	1	0
4. I have felt respected and appreciated.	5	4	3	2	1	0
